# Dissecting Physiopathology of COVID-19

**DOI:** 10.3390/ijms23179602

**Published:** 2022-08-24

**Authors:** Jacek Z. Kubiak, Malgorzata Kloc

**Affiliations:** 1 Dynamics and Mechanics of Epithelia Group, Faculty of Medicine, Institute of Genetics and Development of Rennes, University of Rennes, CNRS, UMR 6290, 35000 Rennes, France; 2 Laboratory of Molecular Oncology and Innovative Therapies, Department of Oncology, Military Institute of Medicine, 04-141 Warsaw, Poland; 3 Transplant Immunology, The Houston Methodist Research Institute, Houston, TX 77030, USA; 4 Department of Surgery, The Houston Methodist Hospital, Houston, TX 77030, USA; 5 Department of Genetics, The University of Texas, MD Anderson Cancer Center, Houston, TX 77030, USA

The COVID-19 pandemic declared on 11 March 2020 by WHO [1] triggered an astounding wave of research on all aspects of this novel viral disease. The pace of research in this unprecedented situation has been remarkable, resulting in the explosion of scientific reports and extraordinary achievements in treatment and prevention. The best example is the number of novel and efficient vaccines created in such short time. The avalanche of research vastly increased our knowledge of SARS-CoV-2 and other coronaviruses. We uncovered and understood some of the hitherto unknown mechanisms involved in the immune response to SARS-CoV-2 infection. Scientific research resulted in novel antiviral drugs and treatments, lowering disease severity and saving human lives. Genetic research identified continuously evolving novel variants of the virus. Epidemiological studies characterized and followed variants’ propagation in various regions of the world. The “Coronavirus Disease (COVID-19): Pathophysiology” Special Issue in *IJMS* covers most of these exciting discoveries.

The COVID-19 pandemic is one of the biggest tragedies in modern world history. However, it forced an unprecedented wave of scientific discoveries. New phenomena were discovered, such as enormous differences in the viral infectivity and course of the disease in children and adults or between different individuals. Although these new observations and research continue to expand our knowledge about this disease, we still have many unanswered questions. Does COVID-19 provoke diabetes? Does it cause orchitis? Why are most children relatively resistant to SARS-CoV-2 infection, while some develop pediatric inflammatory multisystem syndrome (PIMS)? Why do some COVID-19 patients continue to experience symptoms after their initial recovery? These people suffer from the so-called post-COVID-19 syndrome or “long COVID-19”. What causes these long-term effects? Why do some patients, a long time after their purported recovery, suffer from nervous system and brain damage? Another area still not fully understood is the response of different types of immune cells to the initial infection and their role in both the halting and propagation of the virus within the patient body. Additionally, why in only some patients does the immune system goes into overdrive, causing a cytokine storm?

Our Special Issue presents novel research and review papers addressing many of these questions related to COVID-19. The articles included in this Special Issue are focused on prevention, diagnostics, signatures of the disease course, molecules involved, vaccine-related adverse effects, potential treatments, and short- and long-term consequences. There are 3 original research papers [2,3,4] and 11 review articles [5,6,7,8,9,10,11,12,13,14,15].

Clovis Chabert and colleagues [2] identified the Aldo-Keto Reductase 1B10 (AKR1B10) as a key enzyme involved in the expression of proinflammatory cytokines. They came to this conclusion by the transcriptomic analysis of lung samples collected post-mortem from COVID-19 patients. The paper presents pharmacological data on macrophages, supporting the hypothesis of the key role of AKR1B10 in the cytokine storm. Importantly, Chabert and colleagues [2] show that AKR1B10 is secreted, and thus can be exchanged between different cell types. This suggests its involvement in the multi-organ systemic impact of COVID-19 and reinforces its role in the cytokine storm. The authors suggest that pharmacological modulation of AKR1B10 is a potential target for COVID-19 management.

SARS-CoV-2 uses its spike protein (S) to bind to the human cell surface through the receptor angiotensin-converting enzyme 2 (ACE2). Shukla and colleagues [3] identified two ACE2-binding peptoid compounds and developed their dimeric derivatives, called ACE2P1D1 and ACE2P2D1, which blocked the interaction S-ACE2. This resulted in the inhibition of the entry of SARS-CoV-2 pseudovirus—used by the team in their study—into human cells. Importantly, ACE2P1D1 and ACE2P2D1 prevented the infection by a D614G mutant pseudovirus without affecting ACE2 expression and activity, which are important for the blood pressure control. This suggests that these compounds can be safely used in COVID-19 patients in the future.

The article by Passariello and colleagues [4] focuses on the rare adverse effects of vaccines against COVID-19, especially in patients developing a prothrombotic state. They investigated the potential involvement of the Spike protein used in vaccines and/or anti-Spike antibodies in the etiopathogenesis of thrombosis. They focused on structural similarities between the pathogenic Platelet Factor 4 (PF4), related to acute thrombocytopenia and thrombosis, with the Spike protein. They showed that the anti-PF4 antibodies cross-react with the Spike–RBD region and that the PF4 and Spike–RBD proteins interact. The authors suggest that this interaction could result in generation of anti-PF4 antibodies, which could provoke platelets’ aggregation because of the high expression of ACE2 on their surface. This is an important path to follow for understanding the adverse effects of anti-SARS-CoV-2 vaccines.

The review articles in this Special Issue summarise the so-far-accumulated knowledge on different aspects of COVID-19 physiopathology. Paulina Niedźwiedzka-Rystwej and colleagues [5] discuss whether the cytokine storm results from immune system suppression or if it is a positive reaction necessary to clear the virus from the infected organism. The hope of finding new drugs acting against COVID-19 is sustained by the article by Mahdi et al. [6] who present an overview of fluoxetine and fluvoxamine regarding their use in COVID-19. The two drugs are antidepressive agents that were also reported to act as lysosomotropic agents, inhibitors of acid sphingomyelinase in the lysosomes, and ligands of sigma-1 receptors. The authors argue how these actions could be helpful in cases of severe outcomes of COVID-19. Monika Gudowska-Sawczuk and Barbara Mroczko [7] analyze the current knowledge on the role of CXCL10 in SARS-CoV-2 infection. CXCL10 activates its receptor CXCR3 mostly expressed on macrophages, T lymphocytes, dendritic cells, natural killer cells, and B cells. This activation participates in developing the cytokine storm in severe cases of COVID-19. The review convinces that CXCL10 may be a useful predictive biomarker of patient outcome in COVID-19 and that the elevated CXCL10 levels are related to both Acute Respiratory Distress Syndrome (ARDS) and neurological complications in COVID-19. Ashmika Foolchand and colleagues [8] devote their review article to the problem of malnutrition and dietary habits altering the immune system and potentially influencing the course of COVID-19. Intestinal epithelial cells express ACE2 receptors similarly to the respiratory epithelia. Thus, their infection by SARS-CoV-2 provides a link between nutrition, virulence, and the clinical outcomes of COVID-19. In addition, respiratory infection modifies the intestinal microbiota. Altogether, all these factors have an important impact on COVID-19 patients.

The work by Chiara Stassi and colleagues [9] explores the significance of postmortem immunohistochemistry in understanding the pathophysiology of both COVID-19 and COVID-19 vaccine-related adverse rare effects. Maria Narożna and Błażej Rubiś [10], in their article, discuss the necessity of identification of novel strategies and markers as well as diagnostic methods in the fight against the COVID-19 pandemic. They point to a potential alternative approach, which is the analysis of miRNA and the use of miRNA as therapeutics and diagnosis markers. The role of the renin–angiotensin–aldosterone and kinin–kallikrein systems in cardiovascular complications (CVCs) in COVID-19 and long COVID-19 are at the center of interest of the work by Samantha L. Cooper et al. [11]. The main mechanism for the development of CVCs and long COVID-19 is thought to be a consequence of the viral S protein/ACE2 axis, the downregulation of ACE2, and the resulting damage caused by the immune response in the infected organism. Shailendra Pratap Singh and colleagues [12] focus on the role of mitochondrial modulations and autophagy pathways shifts in COVID-19. Indeed, SARS-CoV-2 infection modifies the dynamics of both mitochondria, autophagy, mitophagy, and enzymes regulating the metabolism. The authors discuss the importance of these modifications for the course of COVID-19 and their potential applications as anti-coronavirus therapeutics. Daniela Ricci and colleagues [13] discuss how the innate immune response to SARS-CoV-2 infection influences the adaptive immune response and point out the multiple strategies exploited by SARS-CoV-2 to antagonize the host antiviral response.

The next review article in this Special Issue, by Hee Min Yoo et al. [14], is devoted to the detailed description of diagnostic methods in SARS-CoV-2 identification, including as RT-PCR, digital PCR, and non-PCR-based techniques such as reverse transcription loop-mediated isothermal amplification (RT-LAMP) and reverse transcription recombinase polymerase amplification (RT-RPA). The Special Issue is closed with the review by Changbo Qu and colleagues [15], who ask whether Histamine H1 receptor (H1 receptor) antagonists, known as medications against allergic disease, could be used as therapeutics for COVID-19. They also describe potential mechanisms of H1 receptor antagonists’ action on SARS-CoV-2 and the opportunities for the use of these antagonists in managing COVID-19.

All these articles cover different aspects of COVID-19’s pathophysiology and show new avenues in the understanding of the SARS-CoV-2 infection course and new potential treatments and diagnostic methods.
